# Combined Hepatocellular Cholangiocarcinomas; Analysis of a Large Database

**DOI:** 10.4137/cpath.s500

**Published:** 2008-03-19

**Authors:** Mitchell S. Wachtel, Yan Zhang, Tom Xu, Maurizio Chiriva-Internati, Eldo E Frezza

**Affiliations:** 1 Department of Pathology; 2 Department of Family Medicine; 3 Department of Microbiology and Immunology; 4 Department of Surgery, Texas Tech University Health Sciences Center

**Keywords:** hepatoma, cholangiocarcinoma, combined hepatocellular cholangiocarcinoma, multinomial logit, survival analysis

## Abstract

**Aim:**

Combined hepatocellular cholangiocarcinoma (combined tumor) has been described as either a variant of hepatoma or a variant of cholangiocarcinoma. Prior studies evaluated fewer than 50 patients with combined tumors, precluding multivariate analyses. Posited was the notion that analysis of a large database would yield more definite answers.

**Methods:**

This study used SEER (Surveillance, Epidemiology, and End Results Program of the National Cancer Institute) to analyze 282 combined tumors, 2,035 intrahepatic cholangiocarcinomas, and 19,336 hepatomas between the years 1973–2003. Multinomial logit regression calculated point estimates and 95% confidence intervals (c.i.) for relative risk (rr). Cox regression calculated point estimates and 95% confidence intervals (c.i.) for hazard ratios (ĥ).

**Results:**

Men less often had cholangiocarcinomas than they had combined tumors (rr = 0.63, c.i. = 0.49–0.81). Hepatomas less often than combined tumors presented with distant spread (rr = 0.56, c.i. = 0.43–0.72). Men (rr = 1.50, c.i. = 1.17–1.93) and patients with a known Asian or Pacific birthplace (rr = 2.36, c.i. = 1.56–3.56) more often had hepatomas than they had combined tumors. Among patients not known to have an Asian/Pacific birthplace, a diagnosis of cholangiocarcinoma (ĥ = 0.72, c.i. = 0.63–0.82) or hepatoma (ĥ = 0.75, c.i. = 0.66–0.86) provided a better prognosis than did a diagnosis of combined tumor.

**Conclusion:**

Combined tumors differ from hepatomas and cholangiocarcinomas in terms of distribution and survival patterns in the population; they should be considered neither cholangiocarcinomas nor hepatomas.

## Introduction

Combined hepatocellular cholangiocarcinomas (combined tumors) have been a source of controversy ever since they were described [Allen, 1949]. Multiple studies have been performed [Liver Cancer Study Group of Japan, 1990; Maeda, 1995; Ng, 1998; Jarnagin, 2002; Tickoo, 2002; Tang, 2006], none with more than 50 patients with combined tumors. Some thought combined tumor a form of intrahepatic cholangiocarcinoma [Jarnagin, 2002; Tickoo, 2002]; others felt it to be a variant of hepatoma [Maeda, 1995; Ng, 1998; Tang, 2006]. The Surveillance, Epidemiology, and End Results (SEER) Program of the National Cancer Institute has been found to be an authoritative source of information on cancer incidence and survival in the United States; information concerning this database has been made available at its website (www.seer.com). Posited was the notion that this large database would provide enough patients for multivariate analyses that might better define combined tumors. Assessed were the contributions of gender, stage, age, and place of birth to the distribution of the tumor types. Also evaluated were survival differences among tumor types after gender, place of birth, stage, and age were accounted for.

## Materials and Methods

A case listing session provided patients from the SEER 17 Registry data base over the period 1973–2003. Table 1 displayed inclusion and exclusion criteria and the means by which patient subgroups were created. R 2.5.1 analyzed the data. χ^2^ tests assessed relationships of tumor type and 1) age, 2) stage, 3) birthplace, and 4) gender. Multinomial logit regression compared 1) cholangiocarcinoma and combined tumors and 2) hepatoma and combined tumors with respect to age, stage, birthplace, and gender. The Hausmann test assessed the independence of irrelevant alternatives assumption. The Kaplan-Meier method yielded survival estimates. Log rank tests and Cox regression analyzed survival differences. The Grambsch-Therneau test assessed the proportional hazards assumption. Because frequency and survival analyses were performed, a Bonferroni adjustment was made; null hypotheses were rejected when *P* < 0.025.

## Results

Table 2 displayed frequency distributions for the 22,553 patients. 282 (1.3%) had combined tumors. 2,935 (13.0%) had intrahepatic cholangiocarcinomas. 19,336 (85.7%) had hepatomas. 13,268 (58.8%) lacked distant spread. 5,482 (24.3%) showed distant spread. 6,582 (29.2%) were women. 3,895 (17.3%) had a known Asian/Pacific birthplace. χ^2^ tests showed tumor types differed as regards stage, gender, birthplace, and age (*P* < 0.025, for each analysis).

Multinomial logit regression (Table 3) yielded point estimates and 95% confidence intervals (c.i.) for relative risk (rr). Men were less likely than women (rr = 0.63, c.i. = 0.49–0.81) to develop cholangiocarcinomas than combined tumors. Men more than women (rr = 1.50, 95% c.i. = 1.17–1.93) and patients with known Asian/Pacific birthplaces more than those born elsewhere (rr = 2.36, c.i. = 1.56–3.56) more likely had hepatomas than combined tumors. Hepatomas were less likely to present with distant spread than were combined tumors (rr = 0.56, c.i. = 0.43–0.72).

The Hausmann test was performed. Point estimate vectors for models with (**p_f_**) and without (**p_s_**) cholangiocarcinoma were calculated. Removal of **p_f_** elements related to cholangiocarcinoma yielded a conformable vector (**p_fc_**). The difference vector (**d**) was **d** = **p_s_**–**p_fc_**. Variance-covariance matrixes for models with (**C_f_**) and without (**C_s_**) cholangiocarcinoma were calculated. Removal of **C_f_** elements related to cholangiocarcinoma yielded a conformable matrix (**C_fc_**). The difference matrix (**Q**) was **Q** = **C_s_**–**C_fc_**. Then, χ^2^ = **d^T^Md**, where **M** was the generalized inverse of **Q**. The degrees of freedom (d.f.) was the rank of **Q**. The independence of irrelevant alternatives assumption held (χ^2^ = 0.17, 6 d.f., *P* > 0.50).

Univariate survival analyses (Table 4) showed median survivals ranging from two to six months; log rank tests showed statistically significant differences among tumor types with respect to age, stage, birthplace and gender (*P* < 0.025, for each analysis). Cox regression (Table 5) calculated hazard ratios (ĥ) and 95% confidence intervals (c.i.). The Grambsch-Therneau test showed the proportional hazards assumption did not hold for birthplace (ρ = 0.033; χ^2^ = 20.43, *P* < 0.025); the analysis was stratified by birthplace. Persons with a known Asian/Pacific birthplace lacked statistically significant predictor variables (*P* > 0.025, for each analysis). Among patients without a known Asian/Pacific birthplace, cholangiocarcinomas (ĥ = 0.72, c.i. = 0.63–0.82) and hepatomas (ĥ = 0.75, c.i. = 0.66–0.86) imparted a better prognosis than did combined tumors.

## Discussion

This study compared combined tumors with cholangiocarcinomas and hepatomas. For men more than for women, cholangiocarcinomas were less often seen than were combined tumors. Hepatomas, more so than combined tumors, were seen in men (relative to women), presented without distant spread (relative to presenting without distant spread), and arose in patients with an Asian/Pacific birthplace (relative to those born elsewhere). Among those with a non-Asian/Pacific birthplace, a diagnosis of cholangiocarcinoma or hepatomas conferred a better prognosis than did a diagnosis of combined tumor.

Both hepatitis B and hepatitis C have been found to induce hepatoma [Engstrom, 2003]; recent studies have also implicated both viruses with respect to cholangiocarcinoma [Gatselis, 2007; Hai, 2005; Perumal, 2006; Shaib, 2007]. The pre-S mutant of hepatitis B has been shown to most often be seen in those parts of the world (Asia and the Pacific) that have high rates of hepatoma; a relationship to cholangiocarcinoma has not been found [Fan *y*, 2001; Huy TT, 2003]. This study in part confirmed these results by comparing patients with a known Asian/Pacific birthplace to those without a known Asian/Pacific birthplace. Hepatomas, but not cholangiocarcinomas, were more often seen in persons with an Asian/Pacific birthplace than were combined tumors, when compared with patients born elsewhere.

Limitations of this study included its inability to subclassify combined tumors [Goodman, 1985], to take into account the results of hepatitis virus serologic studies, and to evaluate the effect of alcohol and/or drug abuse. The limitations were more than compensated for by the increased sample size; no prior study of this problem included more than 50 patients, precluding multivariate analyses. The importance of sample size was reflected in this study, which had only 25 combined tumors in patients with an Asian/Pacific birthplace: no survival differences with respect to tumor type were identified among this cohort.

The findings of this study complemented those of a recent summary of laboratory findings. Hepatic progenitor cells can have been shown to be able to differentiate towards the biliary and hepatocyte lineages [Libbrecht, 2006]. The cells have been found to be the source of combined tumors. Most hepatomas, by contrast, have been shown to derive from other cells [Libbrecht, 2006]. Cholangiocarcinomas have not been found to have an origin from these cells [Libbrecht, 2006]. Some animal models suggested a common origin for all three tumors, but this has not been demonstrated in humans [Libbrecht, 2006]. Irrespective of these findings with respect to tumor classification, this study showed that the differences matter little from the patient’s perspective because median survival for all three tumor types is less than six months.

This study showed that combined tumors differ from hepatomas and cholangiocarcinomas in terms of distribution and survival patterns in the population; they should be considered neither hepatomas or cholangiocarcinomas.

## Figures and Tables

**Table 1.** Data acquisition schema.

**Database**
SEER 17 Regs Public-Use, Nov 2005 (1973–2003 varying)
**Selection criteria**
Site: Liver and intrahepatic bile duct
Limited to histologic type:
Cholangiocarcinoma—ICD-0 8160
Hepatoma—ICD-0 8170
Combined tumor—ICD-0 8180
Follow-up: Active
First malignant primary indicator: Yes
Diagnostic confirmation: Microscopically confirmed
Excluded: Autopsy/Death certificate cases
**Data collected and stratification**
Gender
Age at diagnosis: Below median of 65 years and 65 or more years
Stage: No metastases, unstaged, and metastases
Place of birth: Asian/Pacific and other
Survival time in months: Up to 48 months
Vital status

**Table 2.** Frequency distributions (and percents) of 22,553 patients with liver tumors by histologic type with respect to stage, gender, birthplace, and age. For each variables, χ^2^ tests showed the differences with respect to tumor type could not have been explained by chance (P < 0.025).

	**Combined tumors**	**Cholangiocarcinomas**	**Hepatomas**	**Total**
**Stage**				
No metastases	147 (1.1%)	1,376 (10.4%)	11,745 (88.5%)	13,268 (100%)
Unstaged	37 (1.0%)	505 (13.3%)	3,261 (85.7%)	3,803 (100%)
Metastases	98 (1.8%)	1,054 (19.2%)	4,330 (79.0%)	5,482 (100%)
**Gender**				
Women	98 (1.5%)	1,364 (20.7%)	5,120 (77.8%)	6,582 (100%)
Men	184 (1.2%)	1,571 (9.8%)	14,216 (89.0%)	15,971 (100%)
**Birthplace**				
Asian/Pacific	25 (0.6%)	252 (6.5%)	3,618 (92.9%)	3,895 (100%)
Other	257 (1.4%)	2,683 (14.4%)	15,718 (84.2%)	18,658 (100%)
**Age**				
Less than 65 years	146 (1.3%)	1,315 (11.8%)	9709 (86.9%)	11,170 (100%)
65 or more years	136 (1.2%)	1,620 (14.2%)	9627 (84.6%)	11,383 (100%)

**Table 3.** Multinomial logit regression comparing 2,935 patients with intra-hepatic cholangiocarcinomas and 19,336 patients with hepatomas with 282 patients with combined tumors.

	**β (s.e.)**	**Z**	**rr (95% c.i.)**
**Cholangicarcinoma v. combined tumor**			
Intercept	2.4		
Man v. woman	−0.46 (0.13)	−3.53*	0.63 (0.49–0.81)
Unstaged v. no metastases	0.36 (0.19)	1.88	1.43 (0.98–2.09)
Metastases v. no metastases	0.15 (0.14)	1.11	1.16 (0.89–1.52)
Asian or Pacific birth v. other	0.01 (0.22)	0.03	1.01 (0.65–1.55)
65 or more years old v. younger	0.21 (0.13)	1.64	1.23 (0.96–1.58)
**Hepatoma v. combined tumor**			
Intercept	3.92		
Man v. woman	0.41 (0.13)	3.19*	1.50 (1.17–1.93)
Unstaged v. no metastases	0.12 (0.19)	0.65	1.13 (0.78–1.62)
Metastases v. no metastases	−0.58 (0.13)	−4.43*	0.56 (0.43–0.72)
Asian/Pacific birth v. other	0.86 (0.21)	4.08*	2.36 (1.56–3.56)
65 or more years old v. younger	0.11 (0.12)	0.94	1.12 (0.88–1.42)

* *P* < 0.025.

**Table 4.** Univariate survival analyses.

	**Patients n = 22,553**	**Deaths n = 18,803**	**Median survival Months (95% c.i.)**	**Log rank χ^2^ (df)**
**Histologic type**				
Combined	282 (1.3%)	254 (1.4%)	3 (2–3)	
Cholangiocarcinoma	2,935 (13%)	2,568 (13.7%)	4 (4–5)	
Hepatoma	19,336 (85.7%)	15,981 (85%)	4 (4–4)	34.2 (2)*
**Asian or Pacific birthplace**				
No	18,658 (82.7%)	15,645 (83.2%)	4 (4–4)	
Yes	3,895 (17.3%)	3,158 (16.8%)	5 (5–6)	74.4 (1)*
**Age in years**				
65 or more	11,170 (49.5%)	8,872 (47.2%)	5 (4–5)	
64 or less	11,383 (50.5%)	9,931 (52.8%)	3 (3–4)	234 (1)*
**Gender**				
Woman	6,582 (29.2%)	5,435 (28.9%)	5 (4–5)	
Man	15,971 (70.8%)	13,368 (71.1%)	4 (4–4)	43.8 (1)*
**Metastatic status**				
No metastases	13,268 (58.8%)	10,188 (54.2%)	6 (6–6)	
Unstaged	3,803 (16.9%)	3,463 (18.4%)	3 (3–3)	
Metastases	5,482 (24.3%)	5,152 (27.4%)	2 (2–2)	1,991 (2)*

* *P* < 0.025.

**Table 5.** Cox regression analyses.

	**β (s.e.)**	**z**	**ĥ (95% c.i.)**
**Asian or Pacific birthplace (n = 3895)**			
Cholangiocarcinoma v. combined	−0.25 (0.22)	−1.11	0.78 (0.51–1.2)
Hepatoma v. combined	−0.28 (0.21)	−1.34	0.76 (0.5–1.14)
65 or more years v. younger	0.05 (0.04)	1.37	1.05 (0.98–1.13)
Man v. woman	0.14 (0.04)	3.42*	1.15 (1.06–1.25)
Unstaged v. no metastases	0.45 (0.05)	8.51*	1.57 (1.41–1.74)
Metastases v. no metastases	0.79 (0.04)	18.36*	2.21 (2.03–2.4)
**Other birthplace (n = 18658)**			
Cholangiocarcinoma v. combined	−0.33 (0.07)	−4.83*	0.72 (0.63–0.82)
Hepatoma v. combined	−0.28 (0.07)	−4.28*	0.75 (0.66–0.86)
65 or more years v. younger	0.26 (0.02)	16.24*	1.3 (1.26–1.34)
Man v. woman	0.14 (0.02)	7.8*	1.15 (1.11–1.19)
Unstaged v. no metastases	0.41 (0.02)	19.16*	1.51 (1.45–1.57)
Metastases v. no metastases	0.74 (0.02)	38.71*	2.1 (2.03–2.18)

* *P* < 0.025.
